# The Optimal Locomotion of a Self-Propelled Worm Actuated by Two Square Waves

**DOI:** 10.3390/mi8120364

**Published:** 2017-12-16

**Authors:** Ziwang Jiang, Jian Xu

**Affiliations:** 1 School of Aerospace Engineering and Applied Mechanics, Tongji University, Shanghai 200092, China; 0546_jiangzw@tongji.edu.cn; 2 Department of Aeronautics and Astronautics, Fudan University, 220 Handan Road, Shanghai 200433, China

**Keywords:** worm-like locomotion, two square waves, two-segment worm, self-propulsion, optimization

## Abstract

Worm-like locomotion at small scales induced by propagating a series of extensive or contraction waves has exhibited enormous possibilities in reproducing artificial mobile soft robotics. However, the optimal relation between locomotion performance and some important parameters, such as the distance between two adjacent waves, wave width, and body length, is still not clear. To solve this problem, this paper studies the optimal problem of a worm’s motion induced by two peristalsis waves in a viscous medium. Inspired by a worm’s motion, we consider that its body consists of two segments which can perform the respective shape change. Next, a quasi-static model describing the worm-like locomotion is used to investigate the relationship between its average velocity over the period and these parameters. Through the analysis of the relationship among these parameters, we find that there exist four different cases which should be addressed. Correspondingly, the average velocity in each case can be approximately derived. After that, optimization is carried out on each case to maximize the average velocity according to the Kuhn–Tucker Conditions. As a result, the optimal conditions of all of the cases are obtained. Finally, numerical and experimental verifications are carried out to demonstrate the correctness of the obtained results.

## 1. Introduction

Micro-robots, especially worm-like soft robots at a small scale, due to their extensive potential applications in disaster rescue, military detection, pipeline cleaning, medical treatment, etc., have attracted the attention of many scholars. As an example of a micro-robot, a continuous worm-like robot with a one-centimeter diameter, which uses shape memory alloy and a hydrostatic fluid as return spring, is presented [1]. The movement of fluid induced by the sequential constriction of a hoop actuator leads to the generation of peristaltic waves. As a result, the motion of the whole body is naturally caused. However, the optimal design of some parameters, such as body length, wave width, and the distance between two adjacent waves, is still not clear. To this end, this paper deals with the optimal problem of worm-like locomotion driven by two square waves.

The research on these worm-like robots is inspired by the locomotion mechanism of some limbless animals, such as earthworms, snails, caterpillars, and amoeba. Based on the mechanism of the periodic shape change of their bodies, which is induced by the propagation of a series of peristalsis waves, these animals can move in various environments. Compared with other animals with legs or wheels, they are capable of both rapid and dexterous movements in confined complex spaces. In the forward moving process, animals such as snails [2], earthworms [3,4,5], slugs [6], and terrestrial planarians [7] can express multiple peristalsis waves which can be run simultaneously over the length of body. In fact, the number of waves is regarded as a main factor of motion pattern in peristaltic crawling [8,9]. It has been shown that the simultaneous propagation of multiple waves along a worm’s body is possible. However, many researchers have mainly paid attention to the locomotion mechanism of one wave when a worm-like locomotion system was studied [10,11,12,13,14], and did not consider the case of multiple waves.

On the other hand, a trend in the development of a worm-like robot is to increase the number of waves. In fact, a number of waves along the body of a worm-like robot is also possible [1,5,15,16,17,18,19]. A new continuous robot with the exterior braided mesh made of brake cable sheathing was designed. A technique using this braided mesh to produce smooth waves of motion along the body was presented [5]. At the end of this robot there is a cam, the length of which can be adjusted to change the shape of waveform. Additionally, with this style of cam mechanism, any whole number of waves is feasible. Meanwhile, the simulated model revealed that the one-wave model has more slip loss than the two-wave one. This result showed that an increase in the number of waves can reduce the slip backwards. Besides this, a discrete model of a metameric earthworm-like robot has been developed to study the relationship among gait patterns, physical parameters, and dynamic behavior [15]. It is worth noting that the number of peristalsis waves corresponds to the number of driving modules, which are regarded as the locomotion gaits. These gaits can be adjusted to adapt to a changing environment. However, the relationship between worm-like locomotion induced by multiple waves and some important parameters, such as the distance between adjacent waves, wave width, and body length, has rarely been reported.

The average velocity is one of the most important indexes to evaluate the locomotion performance of a worm-like system. The maximum improvement of the average velocity has been an active subject of scientific research. The gait parameters [15] and the phase coordination [20] have been used to study the locomotion of worm-like robots, and the optimal relation of the parameters was obtained to maximize the average velocity. The result revealed that a driving module is the optimal drive mode when the best average progression is attained. In addition, the worm could achieve the maximum crawling velocity by adjusting its tension distribution, and at this time the corresponding wave form was found [21]. The result showed an advantage in the increase of the number of waves along the worm body that can reduce the maximum tension of the body when the worm moves on a rough or sticky surface. Meanwhile, it led to decreases in wave speed and average velocity. However, the interaction force of the aforementioned studies concerning the maximum average velocity is mainly dry friction. After a brief review of this literature, it can be seen that very few studies of the optimal locomotion on parameters such as the distance between adjacent waves and body length have been considered in a linear viscous environment. Therefore, this paper studies the optimal relation between average velocity and these parameters so that the locomotion performance of a self-propelled system can be improved.

In this paper, we consider that worm-like locomotion is simultaneously driven by two square waves with the same travel speed along its body and assume that the worm is able to freely control its shape. As a result, we introduce the case in which the worm consists of two segments, each of which can deform independently [22,23]. Based on the above statement, this paper first explores the correlation between the average velocity of worm-like locomotion over one period and some important parameters by using the quasi-static model. After that, we find the optimal conditions under which the maximum velocity of the locomotion is attained according to the Kuhn–Tucker (KT) Conditions.

The rest of the paper is organized as follows. In Section 2, the reduced dynamic model is introduced to study worm-like locomotion. The square strain waves are utilized to calculate the expressions of the relative velocity and the relative displacement in each interval, and the expressions of average velocities in different cases are calculated by using the reduced model in Section 3. In Section 4, optimization is carried out to obtain the optimal conditions that correspond to the maximum average velocities. Then, the verifications of the optimal conditions and discussion are performed in Section 5. Finally, conclusions are given in Section 6.

## 2. The Mathematical Description of Worm-Like Locomotion

This section is devoted to the description of the model under consideration. In the following, the dynamic model and its reduced model, called the quasi-static model, will be introduced. To establish the relationship between worm-like locomotion and parameters such as the friction coefficient, linear density, wave width, and body length of the worm, a dynamic model of the locomotion [24] has been developed. This model, which describes one-dimensional motion of a worm along a straight line, is denoted by the following form

(1) $${\ddot{x}}_{1}(t)+a(t){\dot{x}}_{1}(t)=b(t)$$

where

(2) $$a(t)=\frac{\mu l(t)}{\rho_{0}L}\;\mathrm{and}\;b(t)=-\frac{\mu }{\rho_{0}L}\int_{0}^{L}\dot{h}(Z,t)h^{\prime }(Z,t)dZ-\frac{1}{L}\int_{0}^{L}\ddot{h}(Z,t)dZ$$

**Figure 1:**
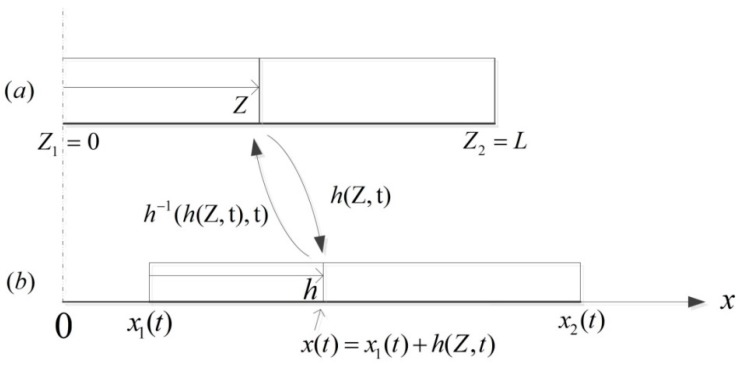
A continuously deforming worm. (**a**) The relaxed body in the reference configuration; (**b**) The deformed body caused by the propagation of strain waves in the current configuration.

Here, Z denotes the coordinate along the worm’s body (shown in Figure 1); $Z_{1}=0,\;Z_{2}=L$ correspond to the rear end and the front end, respectively; L is the body length of the worm in the reference configuration; $x_{1}(t)$ is the coordinate of the left edge of the worm’s body measured from a point fixed in the current configuration and denotes the position of the worm; h(Z,t) is the relative displacement of the point at position Z to the rear end $x_{1}(t)$ and describes the shape of the worm as freely controllable; and l(t)=h(L,t) is the whole length of the deformed body. Here, the prime and the dot represent the derivative of h(Z,t) with respect to the position Z and the time t, respectively. $\rho_{0}$ is the linear density of the worm’s body; μ is the viscous coefficient of the linear friction. This viscous friction is employed in this paper and is given as the following form

(3) f(h,t)=−μv(h,t)

where v(h,t) is the absolute velocity of the locomotion.

It is seen from Figure 1 that the motion of the worm induced by shape change is described by

(4) $$x(Z,t)=x_{1}(t)+h(Z,t)$$

with h(0,t)=0, and $h^{\prime }\left(Z,t\right)>0$, which implies that the adjacent parts cannot pass through each other in the deformation. Mathematically, it can be written as

(5) $$h^{\prime }(Z,t)=1+\epsilon (Z,t)$$

where ϵ(Z,t)>−1, and it is the local strain at the time t and the position Z.

Up to now, as seen from Equation (1), the coefficients of the equation related only to ϵ(z,τ) are determined by a specific strain wave. In the paper, to further obtain the solution of Equation (1), we introduce square strain waves (SSW) as a special type of ϵ(z,τ), which is presented as

(6) $$\epsilon \left(Z,t\right)=\{\begin{array}{ll} {\bar{\epsilon }}_{1}(t), & Z\in [0,\;L_{1})\\ {\bar{\epsilon }}_{2}(t), & Z\in [L_{1},\;L] \end{array}$$

where ${\bar{\epsilon }}_{1}(t)=\epsilon_{1}$, ${\bar{\epsilon }}_{2}(t)=\epsilon_{2}$; $\epsilon_{i}\;(i=1,2)$ is the amplitude of strain wave; and $\epsilon_{2}=\gamma \;\epsilon_{1}\;(\gamma \geq 0)$. $L_{1}$ is the length of the rear segment of the worm’s body with the strain $\epsilon_{1}$. In fact, this type of segmented strain employed has been introduced [22,23].

To simplify Equation (1), the dimensionless variables are given as

(7) $$z=\frac{Z}{L_{0}},\;\tau =\frac{ct}{L_{0}},\;\lambda =\frac{L}{L_{0}},\;\lambda_{1}=\frac{L_{1}}{L_{0}},\;\alpha =\frac{\mu L_{0}}{c\rho_{0}},\;\kappa =\frac{x}{L_{0}}$$

where $L_{0}$ is the wave width, c the wave speed, and x the interval distance between two waves.

Given Equation (7), Equation (1) can be reduced to the following form

(8) $${\ddot{x}}_{1}(\tau )+a_{1}(\tau ){\dot{x}}_{1}(\tau )=b_{1}(\tau )$$

where

(9) $$a_{1}(\tau )=\frac{\alpha }{L}l(\tau )\;\mathrm{and}\;b_{1}(\tau )=-\frac{\alpha }{L}\int_{0}^{\lambda }\dot{h}(z,\tau )h^{\prime }(z,\tau )dz-\frac{1}{\lambda }\int_{0}^{\lambda }\ddot{h}(\tau ,\tau )dz.$$

Further, it is found that Equation (8) can be reduced to a quasi-static approximation for a large friction, a low linear density, a long body length or a small wave speed when the locomotion is driven by the sine-squared strain wave in [24]. Based on the same method, this result can be also obtained by using the drive mode of the square wave. Therefore, this analysis process is omitted but the numerical verification in Section 5 will be given. The reduced result is displayed as

(10) $$\frac{dx_{1}}{d\tau }=-\frac{1}{l_{1}\left(\tau \right)}{\underset{0}{\int }}^{\lambda }\dot{h}\left(z,\tau \right)h^{\prime }\left(z,\tau \right)dz$$

where $l_{1}\left(\tau \right)=L\left(1+\epsilon_{0}q_{1}\left(\tau \right)\right)$ and $q_{1}\left(\tau \right)=\frac{1}{\lambda }\int_{0}^{\lambda }\epsilon (z,\tau )dz$.

Equation (10) is called the quasi-static model, meaning that the inertia effect is ignored. To conveniently analyze the motion of a crawler, this reduced model presented in [12,13,14] is employed in this paper.

It follows from Equations (5) and (7) that the dimensionless relation $h^{\prime }(z,\tau )$ can be given by

(11) $$h^{\prime }(z,\tau )=\frac{L_{0}}{\pi }[1+\epsilon (z,\tau )],$$

where ϵ(z,τ) indicate various strain waves to drive the motion of the worm, for example the sine-squared strain wave [24]. In the following section, we consider ϵ(z,τ) to be a square strain wave (SSW).

## 3. Square Strain Waves (SSW)

The aim of this section is to investigate the relationship between the average velocity of worm-like locomotion and some parameters concerning the body length, the distance between two strain waves, and the strain amplitudes. To this end, we first consider the drive mode of worm-like locomotion.

**Figure 2:**
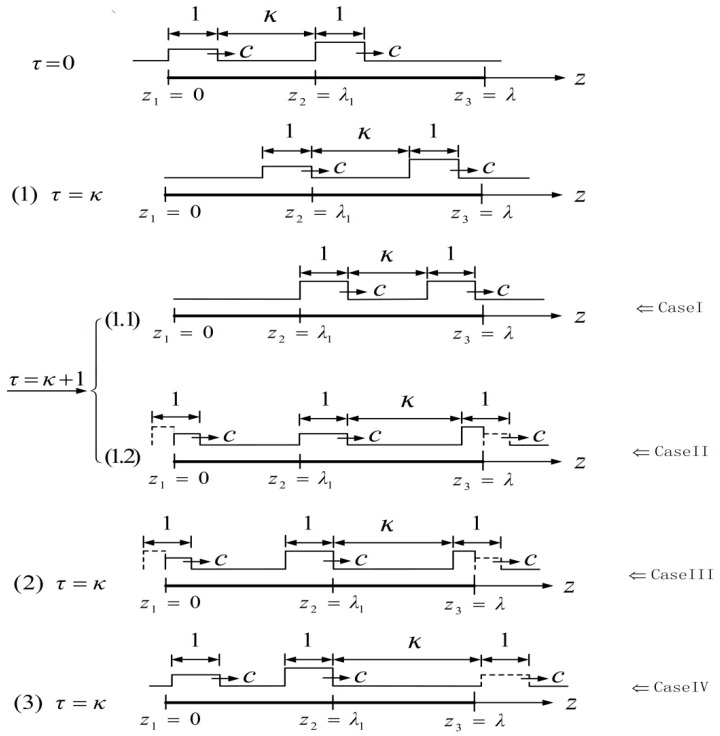
Four cases of worm-like locomotion based on the relationship between the dimensionless body length and the distance of two waves.

In this section, the motion of a worm caused by the simultaneous propagation of two square strain waves with the same travelling speed is typically considered. It should be noted that these two waves may be different when they locate in different positions of the body. Here, square strain waves (SSW) are presented in a dimensionless form in terms of Equations (6) and (7), namely

(12) $$\epsilon \left(z,\tau \right)=\{\begin{array}{ll} {\bar{\epsilon }}_{1}(\tau ), & z\in [0,\;\lambda_{1})\\ {\bar{\epsilon }}_{2}(\tau ), & z\in [\lambda_{1},\;\lambda ] \end{array}$$

where ${\bar{\epsilon }}_{1}(\tau )=\epsilon_{1}$, ${\bar{\epsilon }}_{1}(\tau )=\epsilon_{2}$; here *z* and τ denote the dimensionless position and the dimensionless time, respectively. Meanwhile, the wave width becomes 1, the body length is λ, the wave speed is c and the distance between two waves is κ. These two waves travel rightwards along the worm’s body with the wave speed c and take λ to span the whole body. Because different situations in which these waves locate in different positions in motion will lead to different results, it is found that there are four cases (see Figure 2) which can be addressed in the same way.

Specifically, the first case is that the right wave travels still in the second segment of the worm’s body with the strain $\epsilon_{2}$, while the second case corresponds to that only a certain part of the right wave is located in the second segment of the body when the left wave just passes through the first segment $\left[0,\;\lambda_{1}\right)$ at the dimensionless time τ=κ+1. The third case is that the left wave just reaches the position $z=\lambda_{1}$; meanwhile, one part of the right wave has been out of the interval $\left[\lambda_{1},\lambda \right)$ at τ=κ. However, if the right wave lies outside this interval, this forms the fourth case. In addition, the constraint condition λ≥κ+2 should be satisfied in all of the above cases because the body length is greater than the sum of the width of two waves and the distance between them. For brevity, only the first case is described in detail. The same process for the other three cases is omitted, but these results will be given.

Case I. For λ∈[2κ+3, +∞)

**Figure 3:**
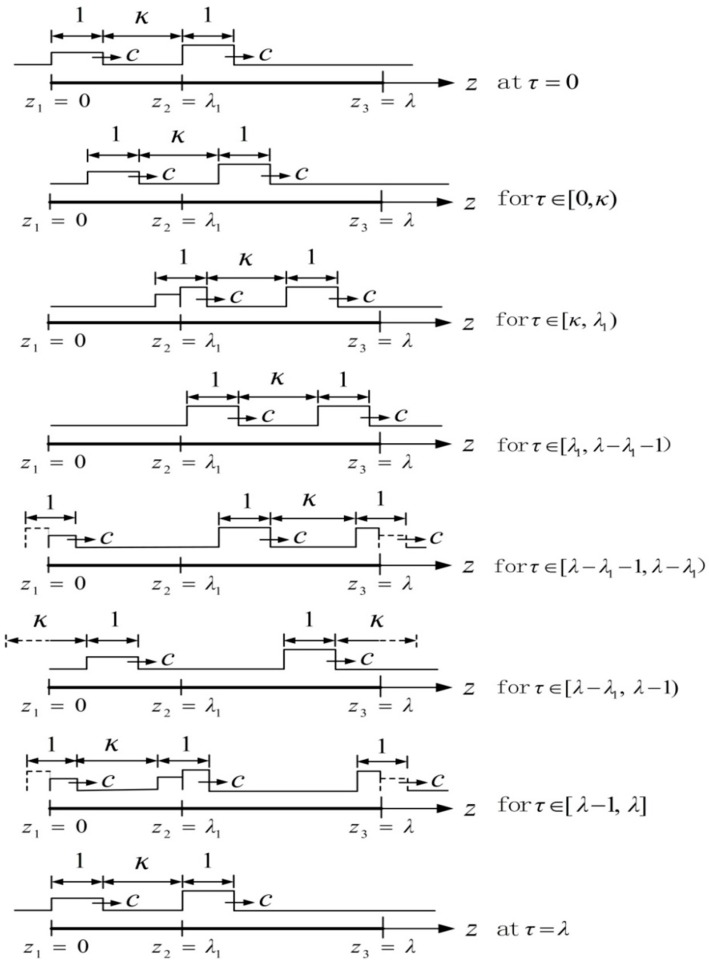
Two square strain waves with a dimensionless unit wave width propagating along the body axis of the worm at the constant speed *c* and the distance between two waves κ

This case is illustrated in Figure 3. When the square wave travels along the interval $\left[0,\;\lambda_{1}\right]$, the strain amplitude of shape change is $\varepsilon_{1}$, while that of another segment is $\varepsilon_{2}$. Six stages take place from the beginning to end in Figure 3.

According to the expression of $h^{\prime }(z,\tau )$ in each interval, the corresponding expressions of h(z,τ) and $\dot{h}(z,\tau )$ are yielded. First, the time-dependent stretch along the worm’s body is given as

(13) $$h^{\prime }(z,\tau )=L_{0}[1+\epsilon_{1}b_{11}(z,\tau )].$$

By integrating Equation (13) with respect to z, one has

(14) $$h(z,\tau )=L_{0}z+L_{0}\epsilon_{1}h_{1}(z,\tau ).$$

Then, the derivative of Equation (14) with respect to τ derives

(15) $${\dot{h}}_{1}(z,\tau )=\epsilon_{1}L_{0}b_{12}(z,\tau ).$$

Using Equation (14), the actual length of the worm is represented as

(16) $$l_{1}(\tau )=L(1+\frac{\epsilon_{1}}{\lambda }q_{1}(\tau )).$$

Here $b_{11}(z,\tau )$ in Equation (13), $h_{1}(z,\tau )$ in Equation (14), $b_{12}(z,\tau )$ in Equation (15), and $q_{1}(\tau )$ in Equation (16) are given in Appendix A due to those complex forms.

Substituting Equations (13), (15) and (16) into Equation (10) and according to the Taylor’s expansion of $1/l_{1}(\tau )$ in the neighborhood of $\epsilon_{1}=0$, one has

(17) $${\dot{x}}_{1}\left(\tau \right)=-\frac{L_{0}}{\lambda }\int_{0}^{\lambda }\left[\epsilon_{1}b_{12}(z,\tau )+{\epsilon_{1}}^{2}\left(b_{11}(z,\tau )b_{12}(z,\tau )-q_{1}(\tau )b_{12}(z,\tau )\right)\right]dz+O({\epsilon_{1}}^{3}).$$

Using Equation (17), the velocity of the left end of the worm’s body ${\dot{x}}_{1j}(\tau )$ for j=1,2,…,6 in the corresponding time interval are calculated and given in Appendix B.

Then, the average velocity of motion over one period can be derived

(18) $$\begin{array}{ll} {\bar{v}}_{1}(\lambda ,\kappa ,\gamma ) & =\frac{\int_{0}^{\kappa }{\dot{x}}_{11}\left(\tau \right)d\tau +\int_{\kappa }^{\lambda_{1}}{\dot{x}}_{12}\left(\tau \right)d\tau +\ldots +\int_{\lambda -1}^{\lambda }{\dot{x}}_{16}\left(\tau \right)d\tau }{\lambda L_{0}/c}\\ & =\frac{c{\epsilon_{1}}^{2}\left(12\gamma^{2}\lambda^{2}+3\left(1+4\kappa +\gamma \left(3-4\gamma \left(3+\kappa \right)\right)\right)\lambda -1-22\gamma +23\gamma^{2}-3\kappa -18\gamma \kappa +21\gamma^{2}\kappa \right)}{6\lambda^{3}} \end{array}.$$

Based on the aforementioned method, the average velocities of the other three cases are obtained and are specifically displayed as follows.

Case II. For λ∈[2κ+2,2κ+3].

(19) $$\begin{array}{ll} {\bar{v}}_{2}(\lambda ,\kappa ,\gamma ) & =\frac{\int_{0}^{\kappa }{\dot{x}}_{21}\left(\tau \right)d\tau +\int_{\kappa }^{\lambda -\lambda_{1}-1}{\dot{x}}_{22}\left(\tau \right)d\tau +\ldots +\int_{\lambda -1}^{\lambda }{\dot{x}}_{26}\left(\tau \right)d\tau }{\lambda L_{0}/c}\\ & =\frac{c\epsilon_{1}{}^{2}}{6\lambda^{3}}\left(\begin{array}{l} \left(\gamma -1\right)\left(2\gamma +1\right)\lambda^{3}+3\left(\left(-3\kappa -1\right)\gamma^{2}+2\gamma \left(2+\kappa \right)+\kappa +1\right)\lambda^{2}\\ +6\left(\gamma^{2}\kappa \left(5+2\kappa \right)-2\gamma \left(1+\kappa \right)\left(3+\kappa \right)+3\kappa +2\right)\lambda -4\gamma^{2}\left(1+\kappa \right)\\ \left(1+\kappa \left(5+\kappa \right)\right)+8\gamma {\left(1+\kappa \right)}^{2}\left(4+\kappa \right)-4\left(\kappa +1\right)\left(\kappa \left(5+\kappa \right)+7\right) \end{array}\right) \end{array}$$

Case III. For λ∈[max{2κ+1,κ+2},2κ+2].

(20) $$\begin{array}{cc} {\bar{v}}_{3}(\lambda ,\kappa ,\gamma ) & =\frac{\int_{0}^{\lambda -\lambda_{1}-1}{\dot{x}}_{31}\left(\tau \right)d\tau +\int_{\lambda -\lambda_{1}-1}^{\kappa }{\dot{x}}_{32}\left(\tau \right)d\tau +\ldots +\int_{\lambda -1}^{\lambda }{\dot{x}}_{36}\left(\tau \right)d\tau }{\lambda L_{0}/c}\\ & =\frac{c\epsilon_{1}{}^{2}}{6\lambda^{3}}\left(\begin{array}{l} \left(-2\gamma^{2}+\gamma +1\right)\lambda^{3}+3\left(\left(3\kappa +5\right)\gamma^{2}-2\kappa \gamma -\kappa -1\right)\lambda^{2}-6\gamma^{2}\left(2\kappa^{2}+3\kappa +4\right)\lambda \\ +6\left(2\gamma \left(\kappa^{2}-1\right)+3\kappa +2\right)\lambda +4\left(\gamma -1\right)\left(\kappa +1\right)\left(\gamma \left(\kappa^{2}-\kappa +1\right)-\kappa^{2}+\kappa +5\right) \end{array}\right) \end{array}$$

Case IV. For λ∈[κ+2,2κ+1].

(21) $$\begin{array}{ll} {\bar{v}}_{4}(\lambda ,\kappa ,\gamma ) & =\frac{\int_{0}^{\lambda -\lambda_{1}-1}{\dot{x}}_{41}\left(\tau \right)d\tau +\int_{\lambda -\lambda_{1}-1}^{\lambda -\lambda_{1}}{\dot{x}}_{42}\left(\tau \right)d\tau +\ldots +\int_{\lambda -1}^{\lambda }{\dot{x}}_{46}\left(\tau \right)d\tau }{\lambda L_{0}/c}\\ & =\frac{c\epsilon_{1}{}^{2}}{6\lambda^{3}}\left(12\gamma^{2}\lambda^{2}-3\left(4\gamma^{2}\left(2+\kappa \right)+3\gamma -4\kappa -3\right)\lambda +\left(3\kappa +5\right)\gamma^{2}+2\gamma \left(9\kappa +7\right)-21\kappa -19\right) \end{array}$$

From the Equations (18)–(21), it can be found that the average velocities obtained in the four cases are quadratic in $\epsilon_{1}$, which is convenient for the optimization in the next section.

## 4. Optimization

In the third section, we have calculated the average velocities of all the possible cases according to the approximate quasi-static model. This section will explore the optimal conditions corresponding to the maximum values of those average velocities under inequality constraints. As can be seen from Equations (18)–(21) and the constraints, the optimized problems in these cases have a uniform form. Therefore, they can be addressed using the same method. In fact, the Kuhn–Tucker (KT) Conditions are an effective method to solve these problems. Details about the KT conditions are omitted here and can be found in [25].

The first case is addressed as follows. The optimized process of this case will be given in detail. To avoid the complex and similar calculations, the similar processes of the other three cases are omitted and only the final results will be displayed.

Case I. For λ∈[2κ+3,+∞]

To make full use of the KT Conditions, Equation (18) is denoted as

(22) $$f_{1}(\lambda ,\kappa ,\gamma )=-{\bar{v}}_{1}(\lambda ,\kappa ,\gamma )$$

Then, the maximum value of ${\bar{v}}_{1}(\lambda ,\kappa ,\gamma )$ is equivalent to solving the minimum value of the function $f_{1}(\lambda ,\kappa ,\gamma )$ under the constraint conditions. That is,

(23) $$\begin{array}{l} \min\;f_{1}(\lambda ,\kappa ,\gamma )\\ s.t.g_{i}(\lambda ,\kappa ,\gamma )\geq 0\;\mathrm{for}\;i=1,2,3. \end{array}$$

where $g_{1}(\lambda ,\kappa ,\gamma )=\lambda -2\kappa -3$, $g_{2}(\lambda ,\kappa ,\gamma )=\kappa$, and $g_{3}(\lambda ,\kappa ,\gamma )=\gamma$.

According to the KT Conditions and Equation (23), one has

(24) $$\begin{array}{l} \nabla f_{1}(\lambda ,\kappa ,\gamma )-\mu_{1}\nabla g_{1}(\lambda ,\kappa ,\gamma )-\mu_{2}\nabla g_{2}(\lambda ,\kappa ,\gamma )-\mu_{3}\nabla g_{3}(\lambda ,\kappa ,\gamma )=0\\ \mu_{1}g_{1}(\lambda ,\kappa ,\gamma )=0,\;\mu_{1}\geq 0\\ \mu_{2}g_{2}(\lambda ,\kappa ,\gamma )=0,\;\mu_{2}\geq 0\\ \mu_{3}g_{3}(\lambda ,\kappa ,\gamma )=0,\;\mu_{3}\geq 0 \end{array}$$

where ∇ is the gradient of the corresponding functions. $\mu_{i}$ for i=1,2,3 are the non-negative constants.

Further, Equation (24) can be reduced to the following form

(25) $$\begin{array}{l} {\left(f_{1}\right)}_{\lambda }-\mu_{1}=0\\ {\left(f_{1}\right)}_{\kappa }+2\mu_{1}-\mu_{2}=0\\ {\left(f_{1}\right)}_{\gamma }-\mu_{3}=0\\ \mu_{1}(\lambda -2\kappa -3)=0\\ \mu_{2}\kappa =0\;\mu_{3}\gamma =0 \end{array}$$

where ${\left(f_{1}\right)}_{\lambda }$, ${\left(f_{1}\right)}_{k}$, and ${\left(f_{1}\right)}_{\gamma }$ denote the derivatives of $f_{1}$ with respect to the variables λ, κ, and γ, respectively.

Next, Equation (25) is specifically solved to derive the optimal values.

Here, one has

(26) $$\frac{\partial f_{1}(\lambda ,\kappa ,\gamma )}{\partial \gamma }=-c{\epsilon_{1}}^{2}(\frac{\gamma (24\lambda (\lambda -\kappa -3)+42\kappa +46)+(9\lambda -18\kappa -22)}{6\lambda^{3}})<0$$

for γ≥0,λ≥2κ+3,κ≥0. It is obvious that the function $f_{1}(\lambda ,\kappa ,\gamma )$ is strictly monotone and decreases with respect to γ. Thus, any solution of the equation ${\left(f_{1}\right)}_{\gamma }-\mu_{3}=0$ in Equation (25) does not exist. Therefore, it is found that the parameters λ and κ can be optimized.

According to Equation (25), one obtains

(27) $$\left\{ \begin{array}{lll} g_{1}(\kappa ,\gamma )=0, & \lambda =2\kappa +3, & \mathrm{for}\;0<\gamma <1;\\ \kappa \in (0,0.5), & \lambda =4, & \mathrm{for}\;\gamma =1;\\ \kappa =0, & \lambda >2\kappa +3, & \mathrm{for}\;\gamma >1, \end{array}\right.$$

where $g_{1}(\kappa ,\gamma )=23-10\gamma -4\kappa \left(1+4\kappa \right)+\gamma^{2}\left(11-4\kappa \left(7+4\kappa \right)\right).$ It can be shown that the optimal body length and the optimal relation related to γ and κ are obtained when γ∈(0,1), i.e., $\epsilon_{1}>\epsilon_{2}$. If γ=1, i.e., $\epsilon_{1}=\epsilon_{2}$, implying that the whole body of the worm performs the same shape changes in locomotion, then the optimal condition is that the optimal body length is four times the wave width and the distance between two waves is less than half a wave width. However, if γ>1, i.e., $\epsilon_{1}<\epsilon_{2}$, at this time the optimal condition is that the distance between two waves is zero and the body length should be greater than the sum of two times the distance between two waves and three times the wave width.

Next, the optimized results of the other three cases are given as follows. The explanations of these results are similar to the ones of the first case and are omitted here. The specific illustration of these cases will be seen in the later discussion of this section.

Case II. For λ∈[2κ+2,2κ+3]

(28) $$\left\{ \begin{array}{lll} \kappa =1, & \lambda =4, & \mathrm{for}\;0<\gamma <1;\\ \kappa \in (1/2,1), & \lambda =4, & \mathrm{for}\;\gamma =1;\\ g_{2}(\kappa ,\gamma )=0, & \lambda =2\kappa +3 & \mathrm{for}\;\gamma >1, \end{array}\right.$$

where $g_{2}(\kappa ,\gamma )=g_{1}(\kappa ,\gamma )$.

Case III. For $\{\begin{array}{ll} \lambda \in [\kappa +2,2\kappa +2], & \kappa \in [0,1]\\ \lambda \in [2\kappa +1,2\kappa +2], & \kappa \in [1,+\infty ) \end{array}$

(29) $$\left\{ \begin{array}{lll} g_{3}(\kappa ,\gamma )=0, & \lambda =2\kappa +1, & \mathrm{for}\;0<\gamma <1;\\ \kappa \in (1,3/2), & \lambda =4, & \mathrm{for}\;\gamma =1;\\ \kappa =1, & \lambda =4, & \mathrm{for}\;\gamma >1, \end{array}\right.$$

where $g_{3}(\kappa ,\gamma )=-23+10\gamma +4\kappa \left(-1+4\kappa \right)+\gamma^{2}\left(-11+4\kappa \left(-7+4\kappa \right)\right)$.

Case IV. For λ∈[κ+2,2κ+1]

(30) $$\left\{ \begin{array}{lll} g_{41}(\kappa ,\gamma )=0, & \lambda =\kappa +2, & \mathrm{for}\;0<\gamma <1;\\ \kappa \in (3/2,2), & \lambda =4, & \mathrm{for}\;\gamma =1;\\ g_{42}(\kappa ,\gamma )=0, & \lambda =2\kappa +1, & \mathrm{for}\;\gamma >1, \end{array}\right.$$

where $g_{41}(\kappa ,\gamma )=-9+4\left(-2+\kappa \right)\kappa +\gamma^{2}\left(3+2\kappa \right)+2\gamma \left(-5+3\kappa \right)$ and $g_{42}(\kappa ,\gamma )=g_{3}(\kappa ,\gamma )$.

**Figure 4:**
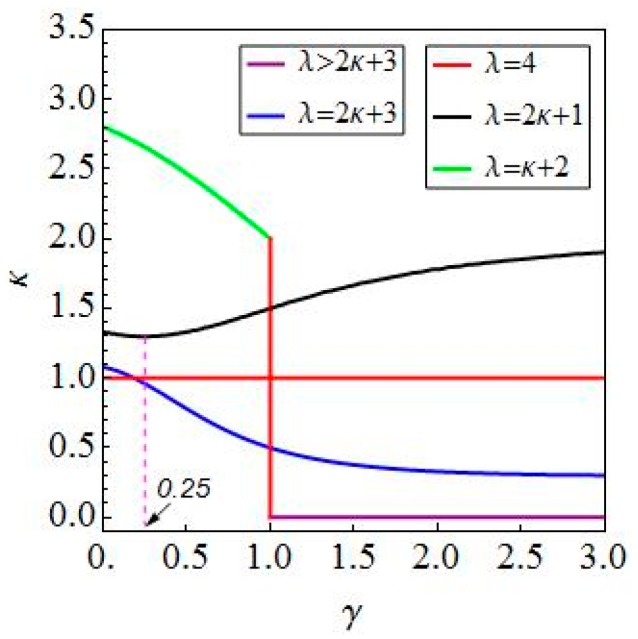
A combination of the optimal conditions for four cases based on the relationship between λ and κ.

The above results reveal that the optimal conditions corresponding to the maximum average velocities are different when the relation between γ and 1 is varied. Meanwhile, the optimized results are depicted in another form in Figure 4.

Next, more comprehensive explanations of these results are given. As can be seen from Figure 4, the optimal conditions for the best average progression have been obtained. To deepen the understanding of the above results, the further discussion is made from the viewpoint of the relationship between $\lambda_{1}$ and $\lambda_{2}$, where $\lambda_{2}=\lambda -\lambda_{1}$. In fact, these two parameters are equivalent to the length of two body segments of the worm.

When $\lambda_{1}<\lambda_{2}$. The relation $g_{1}(\kappa ,\gamma )=0,\;\lambda =2\kappa +3$ for 0<γ≤1 in case I and the same one for γ>1 in case II are denoted by the blue line in Figure 4. It reveals that the distance between two waves is inversely proportional to the ratio of body length to wave width such that the maximum average velocity increases as the distance between two waves decreases along this line. In addition, the other relation κ=0, λ>2κ+3 for γ>1 in case I is also in this situation, which is denoted by the purple line in Figure 4. At this time, the optimal distance between two waves is zero when the maximum average velocity is achieved.

When $\lambda_{1}=\lambda_{2}$. The relation κ=1, λ=4 for 0<γ<1 in case II and the same one for 1<γ<3 in case case III are denoted by the horizontal red line in Figure 4. It means that the optimal distance between two waves is one wave width when the body length is four times the wave width. In addition, the optimal body length is also four times the wave width when γ=1, which is depicted by the red vertical line in Figure 4. However, there is no restriction on the distance between two waves because their maximum average velocities are actually equal. In other words, the number of waves does not affect the worm’s motion at this point.

**Figure 5:**
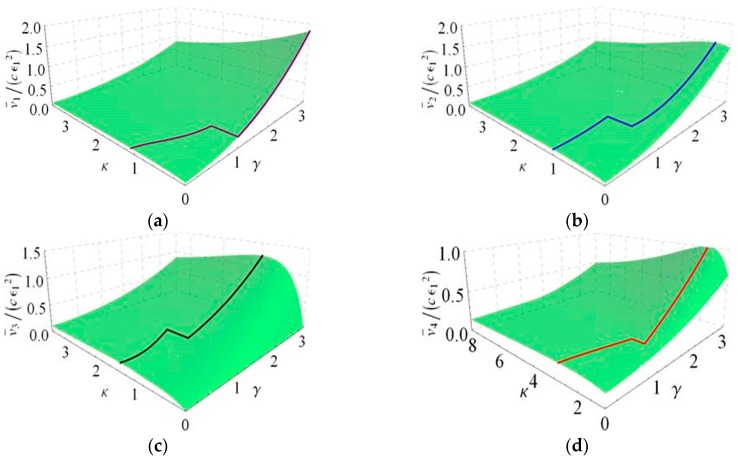
The maximum average velocities related to κ and γ in four cases. (**a**) Case I; (**b**) Case II; (**c**) Case III; (**d**) Case IV.

When $\lambda_{1}>\lambda_{2}$. The relation $g_{3}(\kappa ,\gamma )=0,\;\lambda =2\kappa +1$ for 0<γ<0.25 in case III and the one $g_{41}(\kappa ,\gamma )=0,$ λ=κ+2 for 0<γ<1 in case IV are denoted by the black line and the green line in Figure 4, respectively. It is found that an increase in the distance between two waves leads to a decrease in the maximum average velocity along these two lines. However, the relation for 0.25≤γ≤1 in case III and the same one for γ≥1 in case IV illustrate the opposite result that the distance between two waves is proportional to the maximum average velocity along the black line in Figure 4. Furthermore, it is clearly seen that the optimized result of each case corresponding to the space curve is depicted in Figure 5, which locates in the respective surface obtained by optimizing the average moving velocities ${\bar{v}}_{i}(\lambda ,\kappa ,\gamma )$ for i=1,2,3,4 under various constraint conditions with respect to λ.

## 5. Verification and Discussion

The optimal relations among various parameters in the preceding section have been obtained through the method of KT Conditions. Thereafter, this section will verify the correctness of these results and the reasonableness of the employed model from two perspectives, one is a numerical method, the other is based on former experimental results which have been attained by operating an earthworm-like robot [26].

**Figure 6:**
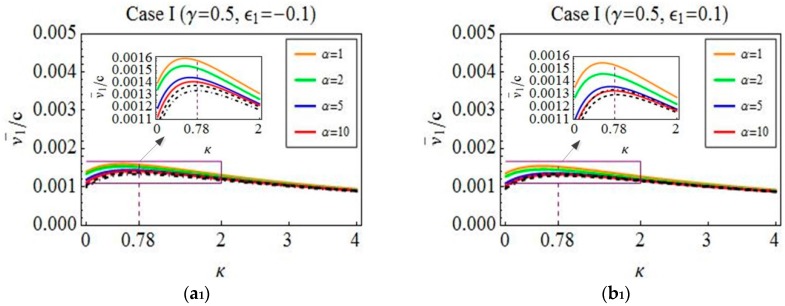

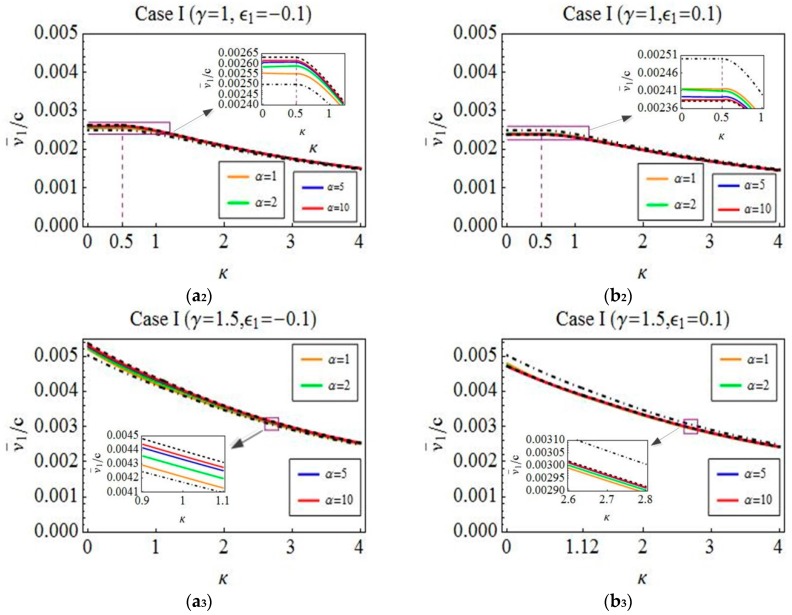
A comparison of three different results in the case of contractive waves (**a**_1_–**a**_3_) and extensive waves (**b**_1_–**b**_3_). The approximate analytical prediction (dotted-dashed line), the numerical solutions from simulating the dynamic equation (solid coloured lines), and the numerical ones from simulating the quasi-static equation (dashed line).

To carry out the numerical verification, some parameters of worm-like locomotion are chosen as γ=0.5,1,1.5, $\epsilon_{1}=-0.1,0.1$, and α=1,2,5,10. Using this verification method and these parameter values, we find that the results on the verified problems of all of the cases are basically similar. Hence, in order to avoid unnecessary repetition, we only consider the verified problems of Case I. Next, the detail explanations on different results in Figure 6 are given. The approximately analytical predictions, which are denoted by dotted dashed lines, are obtained by optimizing the average velocity Equation (18) regarding λ under the given values of γ and $\epsilon_{1}$. Meanwhile, the optimal relation λ=λ(κ,γ) corresponding to the maximum average velocity is derived. Based on this relation and the above-mentioned values γ, $\epsilon_{1}$, and α, the numerical results denoted by dashed lines and solid lines are depicted through the method of simulating the quasi-static equation and the dynamic equation, respectively. A comparison of these three results is done to illustrate the correctness of the theory optimization and the reasonableness of the quasi-static model. More specifically, Figure 6 reveals that the approximate analytical predictions agree well with the numerical results, and the positions of the maximum average velocities are almost the same. It means that the optimized results obtained in Section 4 are correct from this perspective. Additionally, as can be seen from the figure, the average velocities denoted by the solid lines tend to those denoted by the dashed lines when α is increased from 1 to 10, which implies that the dynamic model can be reduced to the quasi-static model for a large friction and a long body length but with a low line density or a small speed.

**Figure 7:**
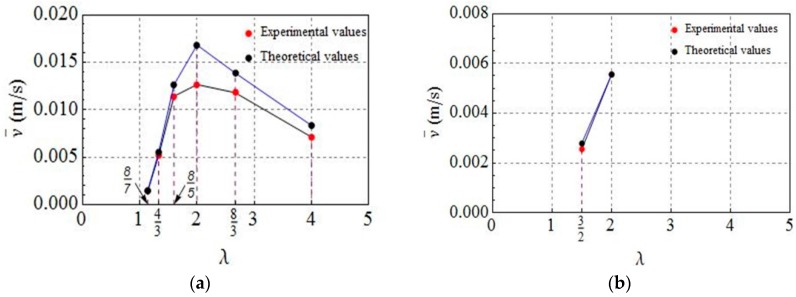
Comparison of experimental results and theoretical results based on the relationship between average velocity and the actually deformed length of the driving modules in [26]. (**a**) One driving module (K=1); (**b**) Two driving modules (K=2).

From another perspective, it is found in [26] that there exist experimental results which can indirectly illustrate one of the optimized results. Based on the analysis of the optimized results of all of the cases, we find that the maximum average velocity is constant when γ=1, λ=4, and κ∈[0,2], which means that the distance between two waves does not affect the worm’s motion when keeping the strain waves along the worm’s body unchanged. At this time, two waves can be considered as one wave, and the optimal wave width is half the body length. It is interesting that the existing experiment can reflect this result as well. Inspired by earthworms’ muscular structure and their locomotion mechanism, Fang et al. firstly developed the general N-segment model of an earthworm-like robot with K driving modules in [26]. Here, the authors considered that the strain waves for driving the worm forward were approximately treated as driving modules, each of which was made up of $n_{A}$ anchoring segments, $n_{R}$ relaxing segments, and $n_{R}$ contracting segments. In fact, their actually deformed segments are $n_{R}$ and $n_{A}$, implying that the ratio of the body length of a robot to the total deformed length of all the driving modules can be denoted by $\lambda =N/(k(n_{A}+n_{R}))$. A group of parameters $\left(K,\;n_{A},\;n_{R}\right)$ was considered as one locomotion gait. The result on the gait optimization corresponding to the maximum average velocity was that: $k=1,n_{A}=1,\;n_{R}=\left[N/2\right]-1$. Then, the sum of $n_{R}$ and $n_{A}$ is [N/2], which means that the optimally deformed length of a driving module is half the body length of the robot. This result is in agreement with the optimal result that the width of two waves is half the length of the worm when r=1. After that, the experimental verification on the optimized results was carried out by designing and operating an eight-segment earthworm-like robot in a horizontal pipe. All of the admissible gaits for this robot were given and employed. The average velocity $\bar{v}$ corresponding to each gait was obtained from a theoretical prediction and an experimental measurement. To clearly illustrate the correctness of the optimal result from the viewpoint of the relationship between $\bar{v}$ and λ, the values of λ corresponding to some representative gaits are selected on the basis of different driving modules K(see Figure 15b–e in [26]). When K=1, we can derive the value $\lambda =\lambda_{1i}$ from the corresponding gait ${\left(n_{A},n_{R}\right)}_{1i}$ for i=1,2,…,6, where $\lambda_{11}=\frac{8}{7}$, $\lambda_{12}=\frac{4}{3}$, $\lambda_{13}=\frac{8}{5}$, $\lambda_{14}=2$, $\lambda_{15}=\frac{8}{3}$, and $\lambda_{16}=4$ and ${\left(n_{A},n_{R}\right)}_{11}=(6,1)$, ${\left(n_{A},n_{R}\right)}_{12}=(4,2)$, ${\left(n_{A},n_{R}\right)}_{13}=(2,3)$, ${\left(n_{A},n_{R}\right)}_{14}=(1,3)$, ${\left(n_{A},n_{R}\right)}_{15}=(1,2)$ and ${\left(n_{A},n_{R}\right)}_{16}=(1,1)$. When K=2, similar to two strain waves, the value $\lambda =\lambda_{2j}$ corresponding to the gait ${\left(n_{A},n_{R}\right)}_{2j}$ for j=1,2 is also solved, where $\lambda_{21}=2$ and $\lambda_{22}=\frac{4}{3}$ and ${\left(n_{A},n_{R}\right)}_{21}=(1,1)$ and ${\left(n_{A},n_{R}\right)}_{22}=(1,2)$. Based on the above statement, both the theoretical and experimental values of the average velocity corresponding to each $\lambda_{kl}$ for k=1, l=1,2,…,6 and k=2, l=1,2 are depicted in Figure 7. As can be seen from this figure, the maximum average velocities are realized when λ=2, which means that the robot can achieve its optimal locomotion performance when the actually deformed length of the driving modules is half that of the earthworm-like robot. To some extent, this result indirectly illustrates that the optimal relations obtained in the preceding section are reasonable. However, this work is still required to be comprehensively verified by designing a continuous worm-like robot from the qualitative and quantitative aspects in the future because here the experimental verification is only based on an approximate earthworm-like robot prototype with relatively few segments. In the other hand, this result can be also verified from the viewpoint of the actual locomotion of the worm [3]. Specifically, it was experimentally observed that the width of a wave of circular contraction is half the body length when the worm moves forward.

The above statement has shown the reasonableness of the optimized results. Therefore, these results are used to discuss the effect of both two waves and one wave on locomotion. Here, one wave is the case that the distance between two waves is zero. As can be seen from Figure 6$\left(a_{3},b_{3}\right)$, one wave is the optimal drive mode by comparison with two waves. Then, in Figure 6$\left(a_{2},b_{2}\right)$, the distance between two waves does not change the value of the maximum average velocity when keeping the body length unchanged. This result shows that the number of waves does not affect the best forward progression of the worm when the motion is induced by square waves. However, it can be seen from Figure 6$\left(a_{1},b_{1}\right)$ that there exists an optimal distance between two waves such that the moving velocity of the worm is improved. For example, in Figure 6$\left(a_{1}\right)$, the value of the average velocity at κ=0.78, λ=4.56, γ=0.5, and $\epsilon_{1}=-0.1$ is derived, which corresponds to the motion generated by two waves. Similarly, the value of the average velocity at κ=0, λ=4.56, γ=0.5, and $\epsilon_{1}=-0.1$ is also calculated, which is the case of one wave. Through a comparison of these two values, the percentage of the improved velocity is 33.24% when the worm’s motion is driven by two waves. The above discussion reveals that the number of waves can affect the locomotion of a worm-like system when the deformation of the worm’s body in locomotion is varied.

## 6. Conclusions

This paper studies the optimization problem of worm-like locomotion induced by two square waves in a linear resistance medium. The relationship between the average velocity and parameters such as the distance between two waves, body length, and wave width is determined by using the reduced dynamic model. Through the optimization of these average locomotion velocities in various situations, the optimal conditions for the fastest average progression are obtained. In the optimal locomotion, there is a noticed case that the whole worm’s body performs the same deformation. At this time, the optimal body length is four times the wave width but the distance between two waves does not affect the locomotion of the worm-like system. The other similar case is that the optimal body length is also four times the wave width, and the optimal distance between two waves is one wave width when the deformation of two segments are different and the length of these two segments is equal. Two specific cases reveal that the effect of the distance between two adjacent waves on locomotion is related to the shape change of a worm’s body and the optimal width of two waves is half the body length. In addition, the results of a numerical simulation and the existing experimental ones from an earthworm-like robot prototype demonstrate the reasonableness of the optimal conditions. Meanwhile, the effect of both two waves and one wave on locomotion is discussed. The results show that the number of waves affects the moving velocity when the shape change of a worm’s body is varied in locomotion. Finally, we hope that these results aid in the deeper understanding of worm-locomotion driven by propagating contractive or extensive waves and provide theoretical support for the design of worm-like soft robots at small scales.

## Appendix A. Expression of $b_{11}(z,\tau ),h_{1}(z,\tau ),q_{1}(\tau )$ and $b_{12}(z,\tau )$

For convenience, the following time intervals are respectively denoted by $I_{j}$ for j=1,2,3,…,6. Namely $I_{1}=[0,\;\kappa )$, $I_{2}=[\kappa ,\;\lambda_{1})$, $I_{3}=[\lambda_{1},\;\lambda -\lambda_{1}-1)$, $I_{4}=[\lambda -\lambda_{1}-1,\;\lambda -\lambda_{1})$, $I_{5}=[\lambda -\lambda_{1},\;\lambda -1)$, $I_{6}=[\lambda -1,\;\lambda )$.

b11(z,τ)={0,z∈[0,τ]1,z∈[τ,τ+1]0,z∈[τ+1,τ+λ1]γ,z∈[τ+λ1,τ+κ+2]0,z∈[τ+κ+2, λ]}if τ∈I10,z∈[0, τ]1,z∈[τ,λ1]γ,z∈[λ1,τ+1]0,z∈[τ+1,τ+λ1]γ,z∈[τ+λ1, τ+λ1+1]0,z∈[τ+λ1+1, λ]}if τ∈I2h1(z,τ)={0,z∈[0, τ]z−τ,z∈[τ,τ+1]1,z∈[τ+1,τ+λ1]γ z−γ(τ+1+κ)+1,z∈[τ+λ1,τ+κ+2]γ+1,z∈[τ+κ+2, λ]}if τ∈I10,z∈[0, τ]z−τ,z∈[τ,λ1]γz−γλ1+λ1−τ,z∈[λ1,τ+1]γ(τ+1−λ1)+λ1−τ,z∈[τ+1,τ+λ1]γz−2γλ1+γ+λ1−τ,z∈[τ+λ1, τ+λ1+1]λ1−τ+γ(2−λ1+τ),z∈[τ+λ1+1, λ]}if τ∈I2b11(z,τ)={0,z∈[0, τ]γ,z∈[τ,τ+1]0,z∈[τ+1,τ+λ1]γ,z∈[τ+λ1,τ+κ+2]0,z∈[τ+κ+2, λ]}if τ∈I31,z∈[0, τ+λ1+1−λ]0,z∈[τ+λ1+1−λ,τ]γ,z∈[τ,τ+1]0,z∈[τ+1,τ+λ1]γ,z∈[τ+λ1, λ]}if ∈I40,z∈[0,τ+λ1−λ]1,z∈[τ+λ1−λ,τ+λ1−λ+1]0,z∈[τ+λ1−λ+1,τ]γ,z∈[τ,τ+1]0,z∈[τ+1, λ]}if ∈I51,z∈[0, τ+1−λ]0,z∈[τ+1−λ,τ+λ1−λ]1,z∈[τ+λ1−λ,λ1]γ,z∈[λ1,τ+λ1+1−λ]0,z∈[τ+λ1+1−λ, τ]γ,z∈[τ, λ]} if ∈I6h1(z,τ)={0,z∈[0, τ]γz−γτ,z∈[τ,τ+1]γ,z∈[τ+1,τ+λ1]γz−γ(τ+λ1−1),z∈[τ+λ1,τ+κ+2]2γ,z∈[τ+κ+2, λ]}if τ∈I3z,z∈[0, τ+λ1+1−λ]τ+λ1+1−λ,z∈[τ+λ1+1−λ,τ]γz−γτ+(τ+λ1+1−λ),z∈[τ,τ+1]γ+(τ+λ1+1−λ),z∈[τ+1,τ+λ1]γ(z−τ−κ)+(τ+λ1+1−λ),z∈[τ+λ1, λ]}if ∈I40,z∈[0,τ+λ1−λ]z−(τ+λ1−λ),z∈[τ+λ1−λ,τ+λ1−λ+1]1,z∈[τ+λ1−λ+1,τ]γz−γτ+1,z∈[τ,τ+1]γ+1,z∈[τ+1, λ]}if∈I5z,z∈[0, τ+1−λ]τ+1−λ,z∈[τ+1−λ,τ+λ1−λ]z−κ,z∈[τ+λ1−λ,λ1]γz−γλ1+λ1−κ,z∈[λ1,τ+λ1+1−λ]γ(τ+1−λ)+λ1−κ,z∈[τ+λ1+1−λ,τ]γ(z+1−λ)+λ1−κ,z∈[τ, λ]}if τ∈I6b12(z,τ)={0,z∈[0, τ]−1,z∈[τ,τ+1]0,z∈[τ+1,τ+λ1]−γ,z∈[τ+λ1,τ+κ+2]0,z∈[τ+κ+2, λ]}if τ∈I10,z∈[0, τ]−1,z∈[τ,λ1]−1,z∈[λ1,τ+1]γ−1,z∈[τ+1,τ+λ1]−1,z∈[τ+λ1,τ+λ1+1]γ−1,z∈[τ+λ1+1, λ]}if τ∈I20,z∈[0, τ]−γ,z∈[τ,τ+1]0,z∈[τ+1,τ+λ1]−γ,z∈[τ+λ1,τ+κ+2]0,z∈[τ+κ+2, λ]}if τ∈I3b12(z,τ)={0,z∈[0, τ+λ1+1−λ]1,z∈[τ+λ1+1−λ,τ]1−γ,z∈[τ,τ+1]1,z∈[τ+1,τ+λ1]1−γ,z∈[τ+λ1, λ]}if∈I4 0,z∈[0,τ+λ1−λ]−1,z∈[τ+λ1−λ,τ+λ1−λ+1]0,z∈[τ+λ1−λ+1,τ]−γ,z∈[τ,τ+1]0,z∈[τ+1, λ]}if∈I50,z∈[0, τ+1−λ]1,z∈[τ+1−λ,τ+λ1−λ]0,z∈[τ+λ1−λ,λ1]0,z∈[λ1,τ+λ1+1−λ]γ,z∈[τ+λ1+1−λ, τ]0,z∈[τ, λ]}if τ∈I6q1(τ)={γ+1,if τ∈I1λ1−τ+γ(2−λ1+τ),if τ∈I22γ,if τ∈I3γ(λ−τ−λ1+1)+τ+λ1+1−λ,if τ∈I4γ+1,if τ∈I5γ−κ+λ1,if τ∈I6

## Appendix B. Expression of ${\dot{x}}_{1j}(\tau )$ for j=1,2,…,5,6

(1) When τ∈[0,κ]
  (31) $${\dot{x}}_{11}\left(\tau \right)=\frac{L_{0}}{\lambda^{2}}\left(\left(1+\gamma +\epsilon_{1}+\gamma^{2}\epsilon_{1}\right)\lambda -\epsilon_{1}\kappa \left({\left(1+\gamma \right)}^{2}\epsilon_{1}\right)\right)$$
  When $\tau \in [\kappa ,\lambda_{1}]$
  (32) $${\dot{x}}_{12}\left(\tau \right)=\frac{L_{0}}{\lambda^{2}}\left(\epsilon_{1}\left(\lambda \left(\lambda -\tau +\gamma \left(2-\lambda +\tau \right)\right)+\epsilon_{1}\left(\gamma \left(\lambda -\tau -2\right)+\tau \right)\left(2\gamma +\left(\gamma -1\right)(\tau -\lambda_{1})\right)\right)\right)$$
(2) When $\tau \in [\lambda_{1},\lambda -\lambda_{1}-1]$
  (33) $${\dot{x}}_{13}\left(\tau \right)=\frac{L_{0}}{\lambda^{2}}\left(2\gamma \epsilon_{1}\left(\lambda +\gamma \left(\lambda -2\right)\epsilon_{1}\right)\right)$$
(3) When $\tau \in [\lambda -\lambda_{1}-1,\lambda -\lambda_{1}]$
  (34) $${\dot{x}}_{14}\left(\tau \right)=\frac{L_{0}\epsilon_{1}}{\lambda^{2}}\left(\begin{array}{l} \lambda \left(1-2\lambda +\tau +\gamma \left(1+\lambda -\tau -\lambda_{1}\right)+\lambda_{1}\right)+\gamma^{2}\epsilon_{1}\left(1+\lambda -\tau -\lambda_{1}\right)\left(\tau +\lambda_{1}-1\right)-\\ \epsilon_{1}\left(2\lambda^{2}-3\lambda \left(1+\tau +\lambda_{1}\right)+{\left(1+\tau +\lambda_{1}\right)}^{2}-2\gamma \left({\left(\tau +\lambda_{1}-\lambda \right)}^{2}-1\right)\right) \end{array}\right)$$
(4) When $\tau \in [\lambda -\lambda_{1},\lambda -1]$
  (35) $${\dot{x}}_{15}\left(\tau \right)=\frac{L_{0}\epsilon_{1}}{\lambda^{2}}\left(\lambda \left(1+\gamma +\epsilon_{1}+\gamma^{2}\epsilon_{1}\right)-{\left(1+\gamma \right)}^{2}\epsilon_{1}\right)$$
(5) When τ∈[λ−1,λ]
  (36) $${\dot{x}}_{16}\left(\tau \right)=\frac{L_{0}}{\lambda^{2}}\left(\epsilon_{1}\left(\kappa +\gamma \left(\lambda -\lambda_{1}-1\right)\right)\left(\epsilon_{1}\left(\gamma -\kappa +\lambda_{1}\right)-\lambda \right)\right)$$
